# New approach methodologies (NAMs) for improved developmental and reproductive toxicity (DART) assessment with focus on endocrine disruption – a PARC project

**DOI:** 10.3389/ftox.2026.1736963

**Published:** 2026-02-03

**Authors:** Jacob Ardenkjær-Skinnerup, Yoni Baert, Lola Bajard, Jonathan Blum, François Brion, Lucia Coppola, Jean-Baptiste Fini, Ellen Fritsche, Henrik Holbech, Selma Hurem, Miriam Naomi Jacobs, Hanna Katarina Lilith Johansson, Dries Knapen, Katharina Koch, Jan Ludvig Lyche, Phuong Ngoc Thi Mai, Winfried Neuhaus, Nikolai Georgiev Nikolov, Atika Nurani, Katerina Papageorgiou, Rikke Poulsen, Louise Ramhøj, Eliška Řehůřková, Anna Kjerstine Rosenmai, Joëlle Rüegg, Iva Sovadinová, Sabrina Tait, Mathew Van de Pette, Tamara Vanhaecke, Lucia Vergauwen, Eva Bay Wedebye, Elvis Genbo Xu, Terje Svingen

**Affiliations:** 1 National Food Institute, Technical University of Denmark, Kongens Lyngby, Denmark; 2 Department of In Vitro Toxicology and Dermato-Cosmetology (IVTD), Vrije Universiteit Brussel, Jette, Belgium; 3 RECETOX, Faculty of Science, Masaryk University, Brno, Czechia; 4 SCAHT - Swiss Centre for Applied Human Toxicology and Department of Pharmaceutical Sciences, University of Basel, Basel, Switzerland; 5 Ecotoxicology of Substances and Environment Unit, National Institute for Industrial Environment and Risks, Verneuil-en-Halatte, France; 6 Center for Gender-Specific Medicine, Italian National Institute of Health, Rome, Italy; 7 PhyMA Laboratory, UMR7221, National Muséum of Natural History, Paris, France; 8 Department of Biology, University of Southern Denmark, Odense, Denmark; 9 Faculty of Veterinary Medicine, Norwegian University of Life Sciences, Ås, Norway; 10 Radiation, Chemical, Climate and Environmental Hazards Directorate, UK Health Security Agency, Chilton, United Kingdom; 11 Department of Veterinary Sciences, University of Antwerp, Antwerp, Belgium; 12 IUF - Leibniz Research Institute for Environmental Medicine, Duesseldorf, Germany; 13 DNTOX GmbH, Duesseldorf, Germany; 14 Competence Unit Molecular Diagnostics, AIT Austrian Institute of Technology GmbH, Vienna, Austria; 15 Faculty of Medicine and Dentistry, Danube Private University (DPU), Krems, Austria; 16 Section for Environmental Chemistry and Physics, University of Copenhagen, Frederiksberg C, Denmark; 17 Department of Organismal Biology, Uppsala University, Uppsala, Sweden

**Keywords:** adverse outcome pathway (AOP), developmental toxicity, endocrine disruption, non-animal methods, regulatory toxicology, reproductive disorders

## Abstract

Endocrine disruptors (EDs) are implicated in adverse developmental and reproductive outcomes, yet their identification remains a major challenge in chemical safety assessment. Current testing strategies rely heavily on animal models, which are constrained by ethical concerns, interspecies differences, and limited mechanistic resolution but justified by the complexity of the endocrine system and its physiology. Capturing the complex biology of intact organisms and incorporating toxicokinetic properties in alternative test methods is challenging. To address this, the European Partnership for the Assessment of Risks from Chemicals (PARC) is advancing the development and regulatory integration of new approach methodologies (NAMs). This project specifically contributes by developing and validating human-relevant NAMs to identify key aspects of endocrine disruption relevant to developmental and reproductive toxicity (DART). Key innovative activities include predictive modeling, refinement of zebrafish and amphibian embryo assays, and establishment of advanced *in vitro* systems for assessing toxicity in the oocyte, testis, placenta, and brain. By combining mechanistic insights with multi-modality and high throughput testing strategies, this work aims to improve the predictive power and regulatory utility of NAMs for ED identification within the One Health paradigm.

## 1 Introduction

Epidemiological and experimental evidence have linked chemical exposures, including endocrine disruptors (EDs), to a range of developmental and reproductive outcomes such as birth defects, altered pubertal timing, reduced fertility, pregnancy complications, and reproductive and neurodevelopmental disorders (Greenspan and Lee, 2018; Skakkebæk et al., 2022; Özel and Rüegg, 2023; Liu et al., 2024; Parent et al., 2025). These outcomes pose serious public health concerns and societal challenges, driving up healthcare costs and contributing to, for instance, fertility rates falling below replacement levels in many industrialized countries (Bellanger et al., 2015; Trasande et al., 2015; Skakkebæk et al., 2022). Effective prevention requires effective identification and regulation of such hazardous substances, yet the identification of EDs in the context of developmental and reproductive toxicity (DART) remains conceptually and methodologically challenging.

**FIGURE 1:**
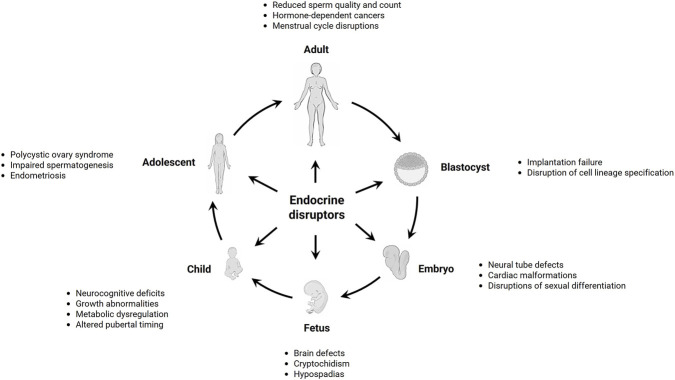
Endocrine disruption may occur at all developmental stages and result in DART-related adverse outcomes, which depend on the window of exposure. Major physiological and anatomical changes occur throughout development, and particularly the prenatal and early postnatal life are sensitive periods. Although examples of adverse outcomes are given at specific life stages, in many instances diseases may also manifest long after actual exposure has occurred. Created with BioRender.com.

DART covers adverse effects of chemical, physical, and biological stressors on the offspring across all life stages, from early embryonic development through fetal growth, postnatal maturation, puberty, and into adulthood. It includes overlapping domains of developmental toxicity (affecting embryonic, fetal, and postnatal life) and reproductive toxicity, which covers developmental toxicity and both male and female aspects of gametogenesis, endocrine regulation, pregnancy, and fertility. A robust DART testing framework is needed to capture a range of hazards, including but not limited to teratogenicity, endocrine disruption, postnatal dysfunction, and transgenerational effects (Jacobs et al., 2017). However, the biological complexity of these processes across life stages and genetic backgrounds, coupled with sex-specific susceptibilities, requires careful considerations of vulnerable windows of exposure when considering key mechanistic endpoints to be assessed (Figure 1).

Despite regulatory progress, assessment of adversity to identify EDs still relies heavily on rodent data (Holmer et al., 2025). This presents several challenges, from practical and economic to ethical. Additionally, animal models may not accurately reflect human biology, particularly in organs such as the placenta or the developing brain, where structural and functional differences between species are pronounced (Florio and Huttner, 2014; Schmidt et al., 2015). To overcome many of these limitations and challenges, efforts are increasingly directed toward developing New Approach Methodologies (NAMs) that are mechanistically anchored, ethically sound, and potentially predictive of effects in humans.

Aligned with OECD test guidelines and the Adverse Outcome Pathway (AOP) framework, alternative methods may offer improved predictive capacity for ED identification (Holmer et al., 2025), while also aligning with ethical and sustainable ambitions of next-generation risk assessment (Petrescu-Mag et al., 2025). Human-based NAMs capable of identifying toxicological mechanisms may also better support the characterization and classification of EDs with human relevance. In parallel, high-throughput early life-stage zebrafish models can provide whole-organism insight and enable rapid screening of many chemicals for early developmental effects of endocrine disruption, complementing mechanism-based, predictive human-relevant assessments (Gölz et al., 2024).

This project, carried out within the EU-funded Partnership for the Assessment of Risks from Chemicals (PARC) program (Marx-Stoelting et al., 2023), aims to contribute toward a mechanism-driven predictive toxicology paradigm rooted in human biology. By integrating NAMs across multiple biological systems and endpoints within DART, the goal is to close critical gaps in available methodologies needed to identify EDs with less reliance on animal models and thereby strengthen the scientific basis for human health risk assessment.

## 2 Background

To advance the identification and characterization of EDs within the context of DART, it is essential to understand both the diversity of endocrine modalities and the mechanistic basis of their disruption. This section briefly outlines the classical and emerging signaling pathways involved in endocrine disruption, the importance of mechanistic approaches, and the challenges and opportunities in extrapolating findings across species to improve the predictive power of human-relevant DART-related NAMs.

### 2.1 Classical and emerging modalities of endocrine disruption

For regulatory ED identification, the focus has historically been on assessing interference with the estrogenic, androgenic, thyroid, and steroidogenesis (EATS) modalities. As the terminology suggests, E refers to endocrine activity related to estrogen receptors (ERs), A to endocrine activity related to the androgen receptor (AR), T to endocrine activity related to the thyroid hormone receptors (THRs), and S to the process of steroid hormone production. These specific modalities remain prioritized due to a relatively good mechanistic understanding and an established battery of OECD test guidelines (e.g., OECD TG456 steroidogenesis assay, OECD TG493 ER binding assay, OECD TG455 ER transactivation assay, and OECD TG458 AR transactivation assay) that are widely accepted in regulatory frameworks, including the U.S. EPA and EU authorities. However, other signaling pathways are increasingly recognized as relevant (OECD, 2012; OECD, 2021).

Non-EATS modalities include disruption to other hormone signaling systems, such as retinoic acid receptor (RAR), retinoid X receptor (RXR), liver X receptor (LXR), peroxisome proliferator-activated receptor (PPAR), aryl hydrocarbon receptor (AhR), and glucocorticoid receptor (GR) signaling. For example, retinoid signaling, mediated by retinoic acid and its target receptors (RAR/RXR), plays essential roles in growth, differentiation, and neurodevelopment, and its disruption can contribute to various DART outcomes (Grignard et al., 2020; OECD, 2021). Similarly, disruption of PPAR signaling has the potential to affect placental-fetal development and function (Adibi et al., 2024), and aberrant regulation has been linked to dysregulated metabolism, angiogenesis, and inflammation in pregnancy-specific disorders (Wieser et al., 2008). AhR, in addition to mediating the metabolism of xenobiotics, is also involved in regulating reproduction and vascular growth; its induction in the placenta can severely impair proper angiogenesis (Li et al., 2020). Furthermore, GR has been implicated in developmental neurotoxicity (DNT), with GR-mediated signaling being important for key neurodevelopmental processes such as neural progenitor cell proliferation and neuronal differentiation, and the progression from progenitor cell proliferation to terminal differentiation (Nürnberg et al., 2018). Additionally, although not an endocrine pathway *per se*, the brain-derived neurotrophic factor (BDNF) pathway, also involved in brain development and function, has been shown to be sensitive to endocrine disruption (Mustieles et al., 2020; Zhao et al., 2020). Likewise, LXR may be relevant in the context of endocrine disruption due to its high expression in the developing brain and regulation of radial glia migration and oligodendrogenesis during corticogenesis both in mouse and human *in vitro* NAMs (Koch et al., 2025). Although these pathways are increasingly recognized as relevant contributors to ED-mediated adverse outcomes (OECD, 2012; OECD, 2021), and *in vitro* assays exist to assess signaling through them, OECD validated test methods for non-EATS mechanisms remain lacking—though some are currently under review.

Based on the many endocrine-relevant signaling pathways linked to DART outcomes, future NAM-based testing strategies should better account for both EATS and non-EATS signaling pathways to ensure comprehensive detection of EDs. Ideally, employed methods should also be capable of transparently detecting multiple mechanisms of action (or key events) within the same assay to minimize the number of NAMs needed to predict individual adverse effects as observed *in vivo*. Within this paradigm, optimizing NAM conditions to simulate the natural environment and hence the specific cell or tissue sensitivity to EDs is also important.

### 2.2 Mechanistic-based approaches in developmental toxicology

Traditional chemical toxicity testing, relying on animal models, is largely observational in that it typically focuses on overt physiological endpoints such as organ weights, malformations, or fertility metrics, without necessarily describing the molecular and cellular perturbations that precede adversities (Browne et al., 2024). In the context of ED assessments, the broadly accepted WHO definition outlines three criteria that must all be fulfilled for ED identification: (i) an adverse outcome in an intact organism, (ii) an endocrine mode of action, and (iii) a plausible causal link between criteria i and ii. This definition of EDs stipulates that mechanisms-of-action must be described (WHO/UNEP et al., 2012), and the specific inclusion of alternative methods has also been consensually agreed (Solecki et al., 2017). Regardless, regulatory ED assessment, for instance for DNT, remains largely restricted to histopathological evaluations and a limited set of behavioral endpoints in rodents. These endpoints do not capture clinically relevant phenotypes of human neurodevelopmental disorders, such as impairments in cognition, language, or social interaction. This limits their effectiveness in detecting early mechanisms of toxicity.

To address this issue, mechanistic approaches in DART testing aim to uncover causal pathways through which chemicals exert their effects, enabling more predictive hazard assessments. By integrating advances in molecular biology, omics technologies, systems toxicology, and computational modeling, these strategies can identify hormone-regulated mechanisms and disrupted signaling pathways, and link stressor events to adverse outcomes. For this, the AOP concept provides a structured framework to capture and organize mechanistic knowledge, promoting mechanism-driven predictive toxicology (Ankley et al., 2010; Audouze et al., 2021).

As the EU advances toward alternative testing strategies, there is a growing need for qualified fit-for-purpose *in vitro* assays that are time- and cost-efficient, while also aiming to cover the full spectrum of potential risks related to DART. Most existing assays typically assess individual endocrine signaling systems, or molecular initiating events, independently of each other, a strategy that is resource-intensive since multiple endocrine systems must be addressed to capture the key molecular events underlying different adverse outcomes.

Developing *in vitro* assays with the capacity to detect multiple mechanisms or endocrine modalities simultaneously, while also being able to identify and quantify pathway perturbations, could improve screening efficiency and better capture biologically relevant crosstalk. For example, estrogen-thyroid interactions influence zebrafish sexual development (Ai et al., 2024), androgen-retinoid crosstalk occurs in human prostate cell lines (Long et al., 2019), and AhR-ER interactions affect reproduction (Tarnow et al., 2019). Recent work has demonstrated that the human Neurosphere Assay, a core component of the OECD initial recommendations on the evaluation of data from the DNT *in vitro* testing battery (DNT IVB) (Blum et al., 2023; OECD, 2023), can detect perturbation of GR, RAR, PPAR, LXR, and RXR signaling (Koch et al., 2025), highlighting its value as a human-relevant NAM that captures multiple endocrine mechanisms in a single assay. Finally, disruption of different endocrine modalities can lead to shared adverse outcomes. For example, anti-androgenic chemicals such as dibutyl phthalate and vinclozolin, as well as anti-thyroid chemicals like amitrole, have all been shown to reduce testis size at birth in rats following intrauterine exposure (Mylchreest et al., 2000; Wolf et al., 2000; Draskau et al., 2024). This complicates predictions using *in vitro* data, especially when considering potential combined effects from simultaneous exposure to multiple chemicals.

### 2.3 Challenges and opportunities in cross-species extrapolation

Endocrine axes are highly conserved across vertebrates, enabling the transfer of knowledge between environmental and human health within a One Health framework. Aquatic models such as the clawed frog (*Xenopus laevis*) and zebrafish (*Danio rerio*) have become valuable beyond ecotoxicology, as effects observed in these model species can signal potential human health risks, emphasizing the interconnectedness of human, animal, and environmental health (Couderq et al., 2020; Exner and Willsey, 2021; Pitt and Gunn, 2024). Their utility in toxicology, neurobiology, and developmental biology stems from their small size, ease of maintenance, high fertility rate, and suitability for high-throughput studies, combined with strong genetic and physiological homology with humans (Szychlinska and Marino Gammazza, 2025). Moreover, these aquatic models can help reduce the use of higher-order vertebrates by serving as intermediates to validate *in vitro* findings before employing rodent studies.

While traditional animal models such as laboratory rodents have provided valuable data on chemical hazards for decades, they are limited by interspecies differences in endocrine physiology, metabolism, and fetal development (Bulun et al., 2005; Gad, 2014). For example, time-matched human and rodent neurospheres display a variety of interspecies differences with regards to physiological and toxicological cues (Baumann et al., 2016; Koch et al., 2025). These interspecies differences underscore the need for human-based *in vitro* models or, as a minimum, a good understanding of when species differences may lead to alterations in mechanistic responses and adverse outcomes.

The placenta represents a key organ displaying marked species differences. It plays a vital role in mammalian fetal development as the primary interface for exchange of oxygen, nutrients, and metabolites between mother and fetus, while also acting as a barrier to fetal exposure. This is an area of concern for regulators, and although attempts have been made to validate *in vitro* placental models within the OECD Test Guideline Programme, these have failed, and further development work is sorely needed. Yet, consistent interspecies differences in placental transfer present major challenges for extrapolation (Schmidt et al., 2021). When also considering placental steroidogenesis and the influence of placenta-derived hormones on fetal development (Mathiesen et al., 2025), the challenges become even more apparent. Hence, to enhance human relevance, *in vitro* approaches using advanced co-culture systems of human cell lines that capture feto-maternal cell-cell interactions offer a promising complement to *in vivo* toxicological studies (Aengenheister et al., 2018; Wong et al., 2020).

## 3 Development of NAMs for endocrine-mediated DART

This project will address critical gaps in identifying EDs relevant to DART by generating mechanistic insights to support the development of NAMs and refining existing methods and AOPs. Aligned with current regulatory needs, activities will address key aspects of DART, including both classical EATS modalities and non-EATS pathways, focusing on their susceptibility to chemical perturbation during sensitive developmental windows. A major objective is the development and refinement of NAMs capable of assessing multiple endocrine modalities within a single assay.

By advancing innovative methods in ED evaluation, the project will primarily facilitate the development of NAMs for ED identification, while also generating DART-relevant, evidence-based mechanistic data to support regulatory decision-making. The project comprises four subprojects addressing specific gaps relevant to ED-mediated DART, as outlined in the following sections.

### 3.1 Subproject 1

**FIGURE 2:**
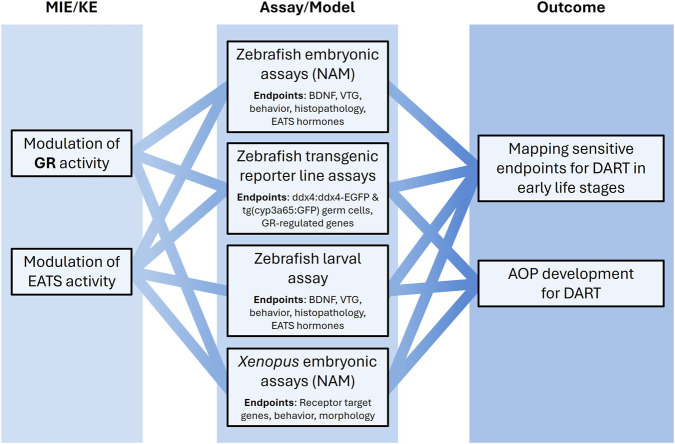
Zebrafish and *Xenopus* embryo-based NAMs will be developed and enhanced for ED identification focusing on multiple ED modalities relevant for DART. Effects in zebrafish eleutheroembryos (NAM model) will be compared to effects in later (non-protected) larval stages of zebrafish. Endpoints include BDNF, vitellogenin (VTG), expression of GR-regulated genes (*fkbp5*, *cyp3a65*), behavior, and multi-organ histopathology. ED identification will be improved via same-sample quantification of steroid and thyroid hormones in zebrafish at early life stages, and transgenic zebrafish reporter lines ddx4:ddx4-EGFP and tg(cyp3a65:GFP) will be employed for direct visualization of germ cell development under chemical exposure and expression of *cyp3a65* in the developing intestine, respectively. In addition, *Xenopus laevis* embryos will be used to assess endocrine axis disruption through morphological, molecular, and behavioral endpoints, providing complementary data for cross-species comparisons and AOP development.

There are ongoing efforts that aim to strengthen and expand the development and validation of zebrafish and *Xenopus* embryo-based NAMs for identifying EDs relevant to both environmental and human health assessment. Because endocrine axes are highly conserved across vertebrates, endocrine-disrupting effects observed in aquatic models may also raise concern for human health (Couderq et al., 2020; Marlatt et al., 2022). Building on previous work (Haigis et al., 2023; Ramhøj et al., 2023), this project will further investigate the cross-species extrapolation potential of these aquatic models (Figure 2).

Validation of a zebrafish embryo test for thyroid hormone system disruption is ongoing under OECD’s validation management group on ecotoxicity testing (VMG-Eco). Aligned with these efforts, we aim to develop a zebrafish embryo test to cover multiple EATS modalities by incorporating endpoints such as vitellogenin gene and protein expression as markers of EAS disruption, BDNF as a biomarker for EATS-related developmental neurotoxicity (Rodríguez-Carrillo et al., 2023), and cognitive behaviors as indicators of adversity. To expand coverage beyond EATS modalities, we will also develop zebrafish embryo-based assays for GR activity. This approach will combine gene expression analysis of GR-regulated genes such as *fkbp5*, which plays an important role in the inhibitory effect of the GR activity *in vivo*, imaging of *cyp3a65* expression using transgenic tg(cyp3a65:GFP) zebrafish embryos (Erradhouani et al., 2025), and mixture modeling approaches. Furthermore, hormone quantification in zebrafish embryos and larvae via LC-MS/MS will be optimized to strengthen ED identification across endocrine axes.

Under the EU Directive 2010/63/EU, zebrafish embryos are considered to be in a non-protected stage until 120 h post-fertilization (≈5 days at 28 °C), prior to reaching the independent feeding larval stage. Comparing effects observed at the (eleuthero)embryo stage (<5 days) with those at later larval stages will help clarify whether early responses are predictive of later-life endocrine-disrupting and neurotoxic effects. This comparison will help answer whether zebrafish embryos can also be used to evaluate adversity induced by one or multiple ED-relevant pathways in addition to assessing the modalities themselves. If so, this would be advantageous in reducing animals in regulatory testing (Knapen et al., 2025).

Disruption of early gonad development and sexual differentiation will be investigated in zebrafish to identify key events for enhancement of relevant AOPs. Studies will assess the effects of chemical exposure during early proliferative and differentiating stages, examining developmental markers from the larval stage through to adulthood. These analyses may also help disentangle toxicological pathways that converge on shared adverse outcomes, distinguishing those driven by endocrine disruption from those arising through other toxicological modalities.

In parallel with the zebrafish models, embryos of the amphibian *Xenopus laevis* will be used to assess the disruption of endocrine axes during early development. *Xenopus* embryos will be exposed to reference EDs and mixtures, followed by analysis of morphological, molecular (target gene expression), and functional (behavioral) parameters to support AOP development. This model will complement zebrafish data by providing a comparative perspective on ED sensitivity in aquatic vertebrates, thereby strengthening the predictive capacity of NAMs for DART effects.

### 3.2 Subproject 2

**FIGURE 3:**
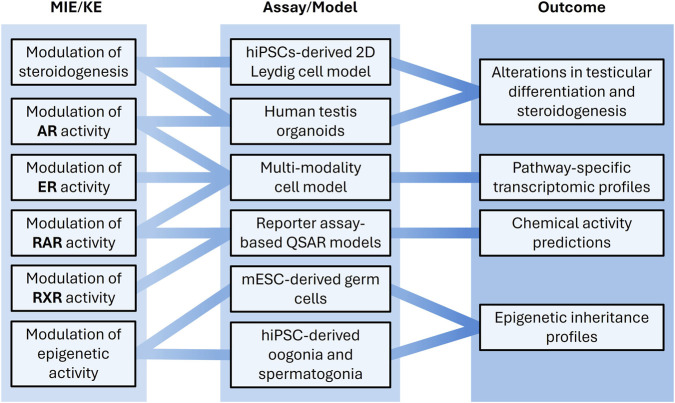
Human-relevant *in vitro* and *in silico* reproductive toxicity models will be developed for improved human health hazard assessment. Molecular initiating events (MIEs) and key events (KEs) related to testicular, germ cell, and receptor-mediated toxicity are linked to advanced model systems and research outcomes, including transcriptomic and epigenetic profiles as well as QSAR model predictions.

The H295R steroidogenesis assay (OECD TG456) is the only validated *in vitro* method for assessing effects on steroidogenesis. However, its adequacy in testing for effects on gonad steroidogenesis is frequently questioned, as it uses a cancerous, adrenal cell line of female origin (Strajhar et al., 2017; Principato et al., 2018). To address this, we aim to develop more physiologically relevant human *in vitro* models that address key limitations of the H295R assay and expand the scope of NAM-based assessment to include multiple endocrine modalities relevant to male reproductive toxicity (Figure 3). Primary human testis organoids (Madureira Silva et al., 2025) will be further optimized and characterized to provide a 3D multicellular system that better captures the structural and functional complexity of the testis. Transcriptomic analyses will be used to generate mechanistic insights into ED effects induced by reference chemicals, with steroidogenesis as the primary focus. Additional relevant modalities, including the androgen modality, will be explored where appropriate. In parallel, human induced pluripotent stem cell (hiPSC)-derived 2D Leydig-like cell models and testis organoids will be refined to study life stage-specific development and sensitivity to ED exposure. Establishing hormone-controlled, chemically defined culture conditions will be explored to enhance physiological relevance and reproducibility by eliminating serum-derived variability and allowing precise modulation of key endocrine signals (e.g., LH, FSH, and testosterone) to better mimic the *in vivo* testicular environment.

A human-relevant cell assay will be developed for multi-modality testing of endocrine disruption via the estrogen, androgen, and retinoid pathways, aiming to identify distinct transcriptional profiles associated with each pathway. The prostate represents a promising model system for this purpose, as all three pathways play critical roles in prostate organogenesis, with their respective receptors expressed at defined developmental stages (Prins and Putz, 2008). To integrate retinoid signaling, predictive quantitative structure-activity relationship (QSAR) models for RAR and RXR agonism will be developed using machine learning methods (Nikolov and Wedebye, 2025). These models will be based on US Tox21 experimental *in vitro* data for approximately 10,000 substances (Richard et al., 2021), with rigorous and transparent data curation following a published procedure (Nikolov et al., 2023). External predictivity and robustness will be evaluated according to the OECD (Q)SAR validation principles.

Although significant progress has been made in the *in vitro* derivation of the germline, human protocols continue to lag behind those established for mice (Yoshino et al., 2021; Murase et al., 2024). Committed oogonia and spermatogonia can now be derived from hiPSCs (Yamashiro et al., 2018; Murase et al., 2024; Nguyen et al., 2024), providing a valuable tool for reproductive toxicity assessment and multi-generational hazard predictions. Transcriptional and epigenetic profiling demonstrate that these cells closely resemble their *in vivo* counterparts; however, their responses to chemical exposure remain largely unexplored. We will use hiPSC-derived spermatogonia and oogonia to study sex-specific epigenetic marks and their responses to selected prototype EDs, thereby establishing proof of concept while further refining differentiation protocols. Additionally, mouse embryonic stem cell (mESC)-derived germ cells will be exposed to EDs to explore species-specific responses of the epigenome to the chemical exposure.

### 3.3 Subproject 3

Currently, neither reproductive/developmental *in vivo* test guidelines nor validated NAMs can adequately assess the impact of EDs on the placenta (Luconi et al., 2022). As an organ, the placenta is sensitive to EDs, with its metabolic and hormonal functions potentially disrupted by ED exposure. EDs may also alter placental vasculature, potentially compromising placental function and, consequently, fetal development (Drwal et al., 2019; Martínez-Razo et al., 2021). The placental barrier primarily consists of a layer of syncytiotrophoblasts (multinucleated placental cells with endocrine activity) and vascular endothelial cells. Disruption of crosstalk between these cell types may impair proper placental vascularization (Myatt and Webster, 2009).

**FIGURE 4:**
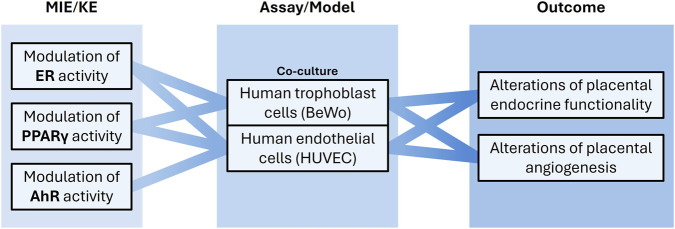
Human-relevant *in vitro* placental toxicity models will be developed to assess endocrine disruptors impacting placental endocrine and angiogenic functions. Molecular initiating events (MIEs) and key events (KEs) associated with receptor-mediated placental toxicity are linked to a co-culture model system that captures placental properties and enables identification of relevant toxicological outcomes.

Building on evidence that some EDs can affect both the endocrine and vascular components of the barrier (Tait et al., 2015; Narciso et al., 2019), a new *in vitro* method will be developed to assess placental angiogenesis by co-culturing relevant human cell lines (Figure 4), replicating the angiogenic and endocrine properties of the human placenta; specifically, human umbilical vein endothelial cells (HUVEC) and human cytotrophoblast (BeWo) cells will be used, the latter being the only line that syncytializes *in vitro*. A panel of angiogenic and endocrine biomarkers will be defined to enhance the utility of the assay for identifying EDs that interfere with placental development. Key endpoints will include the pregnancy hormone β-hCG, the fusogenic protein syncytin-1, and PPARγ for syncytiotrophoblast endocrine function, as well as transcription factors controlling angiogenesis (VEGF and HIF-1α), AhR activity, and vascular tube growth parameters following exposure to prototypical EDs.

### 3.4 Subproject 4

Endocrine disruption in the context of DNT remains a significant regulatory gap, despite compelling evidence from experimental models and epidemiological studies that EDs contribute to adverse neurodevelopmental outcomes. Major efforts within the EURION cluster projects, including ENDpoiNTs (Lupu et al., 2020) and ATHENA (Kortenkamp et al., 2020), have identified a range of molecular pathways implicated in ED-induced DNT, many of which are not adequately addressed by current test methods. These projects have established a portfolio of human and mouse neuronal progenitor cell-derived neuron–glia 2D and 3D models that capture early key neurodevelopmental processes (KNDPs) such as proliferation, differentiation, neurite outgrowth, and migration, which are regulated by well-characterized endocrine mechanisms or modes of action, such as those mediated by glucocorticoids, thyroid hormones, retinoids, fatty acids, and oxysterols (Cediel-Ulloa et al., 2025; Koch et al., 2025).

**FIGURE 5:**
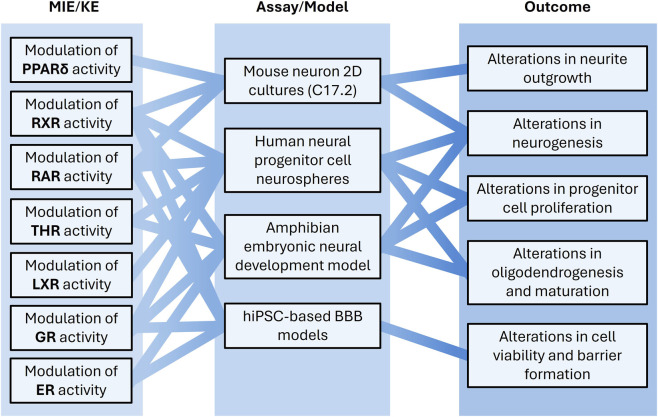
NAM-based testing of ED-induced developmental neurotoxicity will be improved by establishing human-relevant *in vitro* models, enhancing hazard assessment of EDs affecting the endocrine homeostasis during brain development. A *Xenopus laevis* embryonic neural development model provides an additional level of biological relevance for assessing endocrine-dependent processes such as THR, ER, GR, and RXR-mediated pathways. Molecular initiating events (MIEs) and key events (KEs) related to receptor-mediated toxicity are linked to neural model systems, as well as blood-brain barrier (BBB) models capturing multiple KNDPs, which are further linked to cellular outcomes of interest.

Building on these findings, this subproject aims to advance regulatory testing by pre-validating and further characterizing these novel *in vitro* ED-DNT NAMs using multi-omics approaches (Figure 5). The NAM portfolio will be expanded to cover later KNDPs such as neural network formation and function in 3D BrainSpheres (Hartmann et al., 2023), incorporating inflammatory-competent microglia and astrocytes to capture neuroinflammatory processes and their regulation by hormone receptors. These efforts aim to deliver robust ED-DNT assays suitable for regulatory use, covering both early and later stages of human neurodevelopment.

In parallel, multi-omics data from *in vitro* and *in vivo* models, as well as human studies, will be integrated to uncover novel endocrine modes of action, define molecular endpoints, enhance mechanistic insight, increase assay sensitivity, and ultimately reduce reliance on animal testing. To provide an integrated, system-level perspective, the *Xenopus* will be used to investigate neural circuit formation under endocrine control. Immunohistochemical and imaging analyses of neural structures in exposed embryos and tadpoles will be integrated with multi-omics data, thereby providing a complementary approach to support the cellular findings and validate the mechanistic pathways identified in simpler *in vitro* systems.

In addition to neuronal models, the blood-brain barrier (BBB) has been identified by the OECD as a gap for the DNT *in vitro* test battery. BBB *in vitro* models are relevant for studying both the permeability of chemicals into the CNS and their potential to alter barrier function, which can affect communication with brain parenchymal cells such as microglia, astrocytes, oligodendrocytes, and neurons. EDs can modulate BBB function; for example, expression of the tight junction protein Claudin-5 and the efflux transporter ABCG2 (BCRP) are regulated by estrogens and glucocorticoids. Effects of hormones and EDs on the differentiation of hiPSCs into brain capillary endothelial-like cells will be investigated (Appelt-Menzel et al., 2017), based on preliminary data indicating significance of retinoic acid and estrogen for the barrier formation process. Parameters such as cell viability, paracellular barrier integrity, regulation of barrier markers and functionality, and multi-omics-based pathway analysis will be conducted to better understand the effects of EDs on BBB development and validate the applicability domain of the protocols for ED testing.

## 4 Collaboration with other NAM DART initiatives

Regulatory DART testing is mandatory in the EU for pharmaceuticals, plant protection products (pesticides), biocides, and REACH-relevant chemicals produced at >10 tons/year. These studies are resource- and animal-intensive, and species differences may limit direct extrapolation to humans, issues that motivate interest in NAM-based DART assessment among different stakeholders, including academia, industry, and regulators. Two ongoing initiatives in NAM and DART testing strategies will be key collaborators for this PARC project. The first is the Health and Environmental Sciences Institute (HESI)-DART initiative (https://hesiglobal.org/developmental-and-reproductive-toxicology-dart/), which provides a forum for scientists from industry, government, and academia to exchange information, advance DART science, and build consensus on the appropriate use of experimental data for human health risk assessment (Bal-Price et al., 2015). The second is the science-to-policy International STakeholder NETwork (ISTNET)-DART (Fritsche et al., 2024), which aims to support regulatory uptake of NAMs for DART, using the initial recommendations for the DNT NAMs as a model (Bal-Price et al., 2015; OECD, 2023; Blum et al., 2025). This PARC project will collaborate with the existing initiatives to optimize use of funds and avoid duplication of work.

## 5 Conclusion

Aligning with the ongoing shift towards increased reliance on NAM-based testing for chemical safety assessment, such as the European Roadmap to Phasing out Animals in Chemical Testing (EC, 2024) and introduction of new hazard classes for EDs under the CLP regulation (EC, 2023), this project addresses critical gaps in evaluating EDs with DART outcomes. By integrating human-relevant *in vitro* assays (including zebrafish embryos and early larvae, oocyte, testis, placental, and neural models) with predictive computational tools, it aims to deliver sensitive, mechanistically informed NAMs for both EATS and non-EATS modalities. Several of these NAMs have cross-species applicability due to shared upstream mechanisms, enhancing their predictive value and supporting weight of evidence evaluations of key events and complete AOPs. Within the PARC framework, this project will help strengthen regulatory decision-making, promote the adoption of alternative testing strategies, and ultimately improve protection of human health and the environment.
